# Evaluation of the performance of a machine learning based atrial fibrillation screening algorithm using an oscillometric blood pressure monitor

**DOI:** 10.1038/s41598-024-74157-2

**Published:** 2024-10-03

**Authors:** Yuji Asada, Yuta Kudo, Tatsunori Ito, Hiroyuki Kanda

**Affiliations:** Core Technology Department, OMRON HEALTHCARE Co., Ltd., Muko City, Japan

**Keywords:** Atrial fibrillation, Hypertension

## Abstract

Blood pressure monitors (BPMs) with atrial fibrillation (AFib) detection function can be used to detect AFib early. However, conventional algorithms require multiple BP measurements. Here, the feasibility of a machine-learning-based approach for AFib detection through single BP measurement was evaluated. First, a custom AdaBoost-based software, which analyzes the pulse-to-pulse interval (PPI) pattern and classifies it based on AFib detection, was created. Then, its classification performance was validated. For the validation study, PPI and standard 12-lead electrocardiogram (ECG) datasets were collected from 79 and 92 Japanese participants with and without AFib, respectively. PPI data were obtained using two different BPMs. All ECG results were interpreted by cardiologists. The custom software output for the PPI dataset and ECG interpreted results was compared, and the sensitivity and specificity were calculated. A sensitivity and specificity for PPI from main device were 97.5% (95% confidence interval [CI] 91.2–99.3%) and 98.9 (95% CI 94.1–99.8), respectively. No significant differences in sensitivity and specificity were observed in the subgroup analysis between different devices, age groups, and arm size groups. These results reflect the high accuracy and robustness of this AFib algorithm using a single BP measurement and supports its use for widespread AFib screening.

## Introduction

Atrial fibrillation (AFib) is a common arrhythmia that increases the risk of severe strokes^1^. It is an age-related disease, with a prevalence rate of 2.3% among individuals over the age of 40 in the USA^2^. The risk factors for AFib include hypertension, heart failure, chronic kidney disease, and diabetes^3^. Particularly, hypertension and aging increase the risk of AFib^4,5^. With the aging population, the number of AFib cases in the USA^6^ and Japan^7^ is expected to increase in the future. Traditionally, electrocardiogram (ECG) examinations during annual checkup are used to detect AFib. However, in cases of paroxysmal AFib, the symptoms may not manifest during annual checkups. Although some cases of AFib can be detected based on patient-reported symptoms, approximately half of patients with AFib experience no symptoms, leading to undiagnosed AFib.

Ideally, frequent screenings should be performed to effectively detect AFib. Recently, Food and Drug Administration (FDA)-cleared medical devices, including the Apple Watch (Apple Inc., Cupertino, CA) and KardiaMobile (AliveCor Inc., Mountain View, CA), have enabled individuals to self-detect AFib. However, older adult patients who are potentially at higher risk for AFib may not be familiar with these devices. Considering that blood pressure monitors (BPMs) are primarily used by older adults and those with hypertension, they could be integrated with AFib screening functionality, making them an effective AFib detecting tool for targeting high-risk individuals.

Recently, the feasibility of AFib detection using automated BPMs has been reported^8,9^. Wiesel et al. first demonstrated the feasibility of AFib detection based on pulse irregularity from pulse-to-pulse interval (PPI) data collected using BPMs^10^. Since then, several studies have continuously improved the algorithm and conducted clinical evaluations to show the efficacy of AFib screening using BPMs^11–17^. However, conventional algorithms require multiple time measurements to achieve high sensitivity and specificity for AFib detection. Multiple-time BP measurements has potential issues such as longer measurement times and the requirement for the user to switch to a special measurement mode to detect AFib. Hence, many users may not use the AFib measurement mode despite its availability. Particularly, it is thought that asymptomatic individuals are less likely to use the AFib detection mode on BPMs. Moreover, Senoo et al.^18^ reported that 37.7% of patients with AFib are asymptomatic. Screening could be done unintentionally if a single-time measurement could be realized, and many of these asymptomatic individuals are hoped to be screened early.

In this study, we investigated the possibility of detecting AFib with a single measurement by introducing machine learning technology to achieve single-time BP measurement. Hence, we developed a novel algorithm for AFib detection using machine learning technology with high sensitivity and specificity. We evaluated the performance of our algorithm several times during development using heartbeat data created by open source ECG dataset. However, its actual accuracy for PPI in real clinical settings remains unknown as machine learning technology potentially increases the risk of data overfitting for a limited number of datasets.

Therefore, in this study, we aimed to validate the accuracy of the AFib algorithm using a clinical PPI dataset. Accordingly, we performed a clinical study to obtain the PPI dataset of participants with and without AFib who were recruited from multiple clinical sites in Japan.

## Methods

### AFib detection algorithm

Oscillometric BPMs detect the pressure pulse waves (PPWs) or oscillations of the arterial pulse during its BP measurement. The PPWs are then used to calculate PPIs and pulse amplitudes. These PPIs and pulse amplitudes are subsequently used to determine systolic BP, diastolic BP, and pulse rate.

**Fig. 1:**
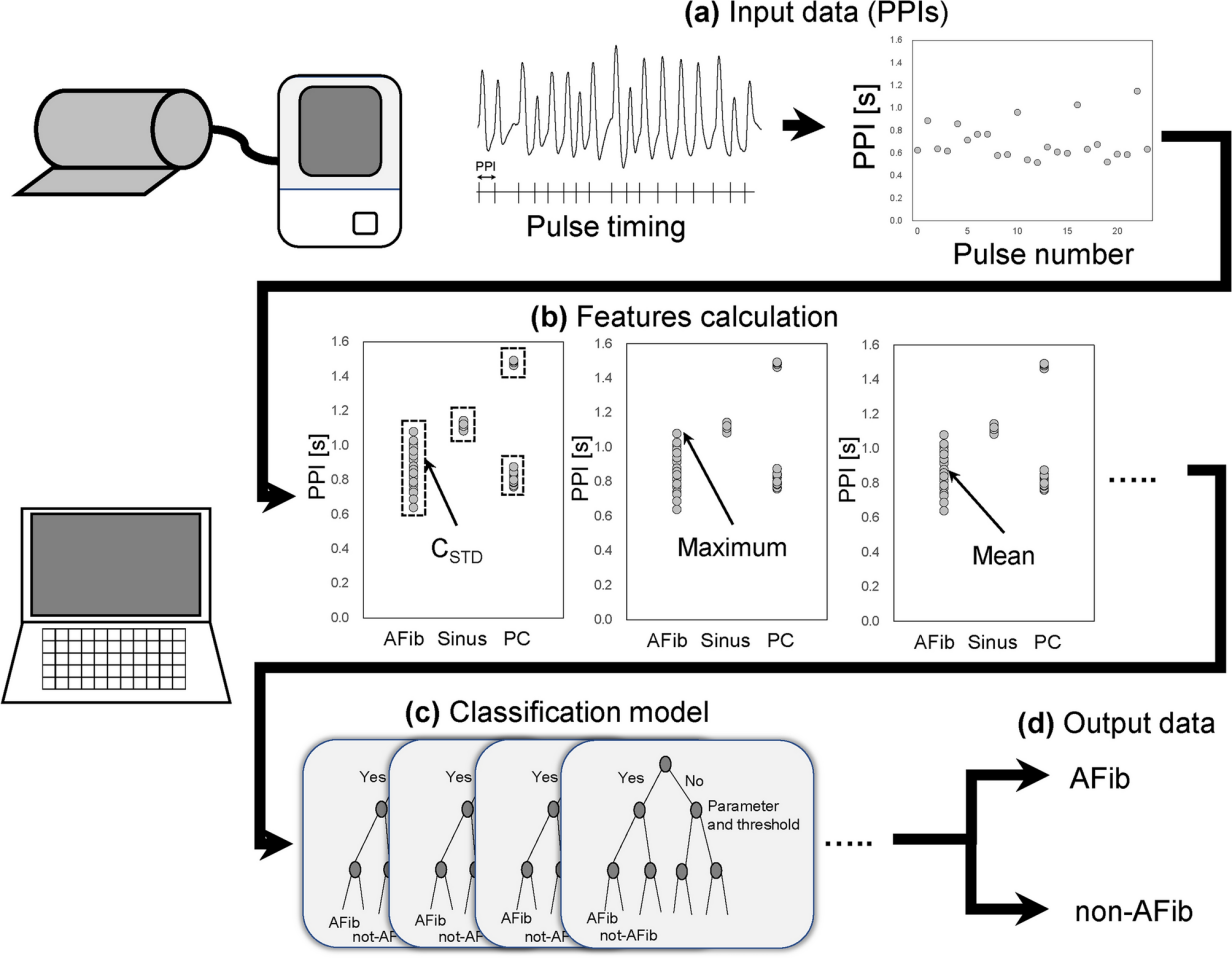
Overview of the AFib detection algorithm. The AFib detection algorithm captures pulse-to-pulse intervals (PPIs) (**a**) as input. The PPIs are converted into feature characteristics (such as C_STD_, maximum, and mean) (**b**). C_STD_ is the calculation of the standard deviation of clustered PPIs. For example, PPIs of non-AFib such as sinus rhythm are grouped together into a single small cluster. PPIs of non-AFib arrhythmias, such as premature contractions (PCs), fluctuate and are also divided into small clusters. Conversely, PPIs in AFib vary widely and randomly, resulting in a single broad cluster (**b**). AdaBoost-based classifiers receive feature characteristics as input (**c**). The classifier outputs AFib versus non-AFib (**d**).

Our proposed algorithm for detecting AFib employs PPI data computed using oscillometric BPMs (Fig. 1a) (patent publication number: WO/2024/122104). This AFib algorithm is unique as it uses a single BP measurement to obtain the PPI data. Patients with AFib are known to have irregularly irregular heartbeat patterns^3^. Conversely, normal sinus rhythm will demonstrate a more consistent pulse interval (see Results section). Additionally, in premature contractions (PCs), which is the most common arrhythmia, patients usually have the same heartbeat pattern and not always a random pattern. Thus, we believe that distinguishing PPIs between random and nonrandom patterns through all PPIs is important for detecting AFib PPIs.

The AFib screening algorithm is composed of a features calculation (Fig. 1b), a classification model (AdaBoost model) (Fig. 1c), and output classification result as AFib or non-AFib (Fig. 1d). The features calculation is comprised of numerous statistical parameters used to both calculate and express the distribution of PPIs. This data is then used in the classification model. The statistical parameters were selected by using “predictorImportance” function of Statistics and Machine Learning Toolbox 11.5 in MATLAB 2019a (The MathWorks Inc., Natick, MA, https://matlab.mathworks.com/) environment from numerous available options for common statistical parameters software. The primary selected statistical parameters included the statistical average, maximum value, percentiles, and standard deviation of PPI distribution (Fig. 1b). In the standard deviation calculation, we used Clustered Standard Deviation (C_STD_). The C_STD_ is a type of standard deviation designed by Plesinger et.al.^19^ to distinguish AFib from sinus rhythm as well as from other arrhythmias. In the C_STD_ calculation, standard deviation is calculated from the squared deviation from the mean of each clustered PPI distribution, instead of using the mean of the whole PPI distribution^19^.

Among these features, C_STD_ showed the most important characteristic in our algorithm using “predictorImportance” function of Statistics and Machine Learning Toolbox 11.5 in MATLAB 2019a. Conversely, we confirmed that removing parameters with low importance in the function (e.g., kurtosis) from the algorithm showed little effect on accuracy. Thus, we excluded them from the algorithm to reduce the data volume.

Recently, several studies have demonstrated that machine learning algorithm results in more accurate judgments compared with cardiologists^20^ or traditional rule-based methods^21^. We investigated the machine learning approach, “AdaBoost classifier”^22^, to build the classification model in this study (Fig. 1c). AdaBoost is an ensemble learning algorithm that enhances prediction accuracy by iteratively combining multiple weak learners including simple decision trees, in a series where each subsequent learner prioritizes correcting the errors from those prior. This method effectively builds a cumulative strength in the model, leading to improved accuracy. The AdaBoost based classification model consists of multiple decision trees using statistical parameters as input for decision trees (Fig. 1c). The classification model was developed and trained using the “AdaBoost.M1” function of the Statistics and Machine Learning Toolbox 11.5 in MATLAB 2019a.

Random (AFib) was distinguished from nonrandom (non-AFib) PPI patterns in the classification model by training it with the input of more than five thousand heartbeat data points retrieved from the ECG databases: MIT-BIH Atrial Fibrillation Database^23^ and PhysioNet/Computing in Cardiology Challenge 2017 Database^24^. The training dataset mainly comprised AFib, normal sinus rhythm, and PC.

Moreover, we aimed to develop a custom software that could be efficiently implemented on BPM devices. This goal was facilitated by our conversion of the MATLAB-based software to C language-based software by using MATLAB Coder 4.2 and Embedded Coder 7.2 (The MathWorks Inc., Natick, MA, https://matlab.mathworks.com/). However, in this study, our custom AFib detection algorithm (Version 1.02) was run on a personal computer in our laboratory, not a microcomputer in a BPM device.

### Evaluation for accuracy extracting PPI from PPW

In order to evaluate for accuracy extracting PPI from PPW in the BPMs, we collected PPI data under the artificial PPW of precisely constant PPI using a noninvasive BP simulator CuffLink (Fluke Biomedical, Inc., Everett, WA). CuffLink is a device that generates artificial PPW under set conditions. In this study, CuffLink settings were set at constant PPI of 0.75 s (= pulse rate 80 bpm) with normal sinus rhythm. Measurements were repeated 30 times each using an HCR-7501T main unit and HEM-FM31 cuff, and HEM7342T-Z main unit and HEM-FL31 (OMRON HEALTHCARE, Muko City, Japan). Average and standard deviation of PPIs in each measurement were calculated.

### Population for collecting PPI and ECG datasets

We conducted a clinical study to create a new PPI dataset for validation of the algorithm. The study protocol followed the guidelines of the Declaration of Helsinki and was approved by the ethical review boards of all participating institutions (No. IRB-2154, approved by OMRON HEALTHCARE IRB committee).

We collected PPI data from participants with and without AFib history and classified them into AFib and non-AFib groups, respectively. For the AFib group, we recruited participants who met the following inclusion criteria: (1) AFib history, (2) AFib findings confirmed by ECG on the day of the test, (3) upper arm circumference between 22 and 36 cm, and (4) age of at least 22 years. Participants were excluded if they were (1) pregnant, (2) had a mastectomy (women only), (3) had pacemakers and/or defibrillators, or (4) exhibited difficulty measuring BP or ECG in the sitting position. Participants in the non-AFib group were recruited based on the following criteria: (1) no AFib records, (2) no AFib findings on ECG on the day of the test, (3) upper arm circumference between 22 and 36 cm, and (4) age of at least 22 years. The exclusion criteria for the non-AFib group were the same as those for the AFib group. A pre-ECG measurement was performed immediately before the main measurement to confirm the presence or absence of AFib findings in the AFib group and non-AFib group, respectively. Additionally, the upper arm circumference of each participant was measured to ensure it was within the inclusion criteria range. All participants provided written informed consents upon enrollment.

Participants for the non-AFib group were primarily recruited in a controlled manner, with age being the primary factor. First, individuals aged 65 years and over were prioritized for recruitment, aiming to recruit approximately 50% of the total non-AFib group from this age group. This was done to ensure that the age distribution of the non-AFib group was similar to that of the AFib group. Second, individuals under the age of 65 years were recruited for the non-AFib group, with equal representation of participants in their 20s, 30s, 40s, and 50s. This was done to ensure that the data collected reflected a diverse population, including younger and older adults. Age-controlled recruitment was only performed in the non-AFib group because of the difficulty of recruiting younger individuals in the AFib group.

Participants were recruited, and data were collected at the following three facilities in Japan: Koseikai Takeda Hospital; Doctors Inc.; and OMRON HEALTHCARE Co., Ltd. Data collection and participant recruitment for the AFib group were primarily conducted at Koseikai Takeda Hospital and Doctors Inc.

### Devices for PPI and ECG data collection

Two BPM devices were used to collect PPI data: the HCR-7501T main unit and HEM-FM31 cuff (OMRON HEALTHCARE, Muko City, Japan), and the HEM7342T-Z main unit and HEM-FL31 cuff (OMRON HEALTHCARE). The HCR-7501T main unit and HEM-FM31 cuff were abbreviated as “FM31,” whereas the HEM7342T-Z main unit and HEM-FL31 cuff were abbreviated as “FL31.” Both devices differ only in cuff size. The HEM-FL31 cuff is used for arm circumferences of 22–42 cm, whereas the HEM-FM31 cuff is used for arm circumferences of 17–36 cm. FM31 was used as a main-device and FL31 was used as a sub-device in this study. The BPM devices were equipped with a custom mode to transmit measurement results, including PPIs, date, and time, to a personal computer via a USB cable. A 12-lead ECG device (ECG Explore 500X1; SAN-EI MEDISYS Co., Ltd., Kyoto, Japan) was used to measure ECG data.

### Procedure for PPI and ECG data collection

The main measurement involved the simultaneous collection of ECG and BPM data. ECG was measured in a seated, rather than a supine, position to comply with BPM guidelines^25^. However, this may increase the risk of myoelectric noise contamination in the ECG signal. Thus, the Manson-Likar method^26^ was used to install the ECG electrodes in order to reduce myoelectric noise interference. After collecting PPI and ECG data, cardiologists interpreted each ECG data obtained during BP measurement and classified it as AFib, non-AFib, or unreadable.

All participants underwent BPM measurements starting with FL31 to FM31. In principle, each participant had one BPM measurement from each device. However, these BPMs have various error detection functions, and if they detect body movement or abnormalities of pulse rate during blood pressure measurement, they will display an error and not display the blood pressure value. There was no additional error processing specific for the AFib detection function, and the dataset when BPM errors were not displayed was used for analysis. Up to two repeated measurements were performed if the BPM displayed an error. We only used the last recorded data for the validation dataset. The entire PPI dataset with each corresponding ECG interpretation was stored within our database.

The association between the R-R interval (RRI) on the ECG and the PPI detected by the BPM device was analyzed 94 intervals dataset from the last five participants with AFib in the same way as reported by Ishizawa et al.^16^ and Kabutoya et al.^17^.

### Calculation of sensitivity and specificity

The custom AFib algorithm software on a personal computer was used to generate output for the PPI dataset in order to assess the algorithm’s sensitivity and specificity. The custom software output a classification result indicating the presence or absence of AFib. Next, the software output and ECG interpretation results were compared to calculate the sensitivity and specificity of the AFib algorithm. The following equations were used to estimate sensitivity and specificity:

$$Sensitivity = \frac{Number\, of \,PPIs \,with\, AFib \,ECG\, that \,were\, output \,as \,AFib\, by\, BPM }{Total \,number\, of \,PPIs \,with \,AFib\, ECG}$$

$$Specificity = \frac{Number \,of\, PPIs\, with \,nonAFib \,ECG\, that\, were\, output\, as\, absence\, of\, AFib\, by\, BPM }{Total \,number\, of\, PPIs\, with\, non\, AFib\, ECG}$$

### Statistical analysis

First, the performance of the AFib detection algorithm on the PPI dataset from the main-device “FM31” was mainly assessed by calculating the sensitivity and specificity values, with associated two-sided 95% confidence intervals (CIs). Then, same analysis was performed on PPI dataset from sub-device “FL31.” The McNemar test was used to determine whether there was a statistically significant difference in the performance between the two cuff types (FM31 and FL31 datasets).

A subgroup analysis based on age and upper arm circumference was conducted to investigate the impact of individual differences on sensitivity and specificity within the FM31 dataset. First, the PPI dataset in FM31 was stratified into two age groups (< 65 and ≥ 65 years) and two upper arm circumference groups (< 27 and ≥ 27 cm). The classification of arm circumference was decided by median of arm circumference (27 cm) of the all participants. Then, the PPI data for each subgroup were analyzed. A chi-square test was performed for the subgroup analysis. All statistical analyses were performed using JMP15.1.0 statistical software (SAS Institute Inc., Cary, NC, http://www.jmp.com).

## Results

### Accuracy of extracting PPI

We evaluated the accuracy of extracting PPI by using the CuffLink PPW simulator under constant PPI condition. Using the FM31 and FL31, the average of measured PPI were 0.75 ± 0.00 s and 0.75 ± 0.00 s (mean ± SD) respectively, which adequately measured the CuffLink setting of 0.75 s. The mean standard deviation of PPI measurements using the FM31 and FL31 were 10.8 ± 2.1 ms and 14.5 ± 3.4 ms, respectively.

### Participants’ background

**Fig. 2:**
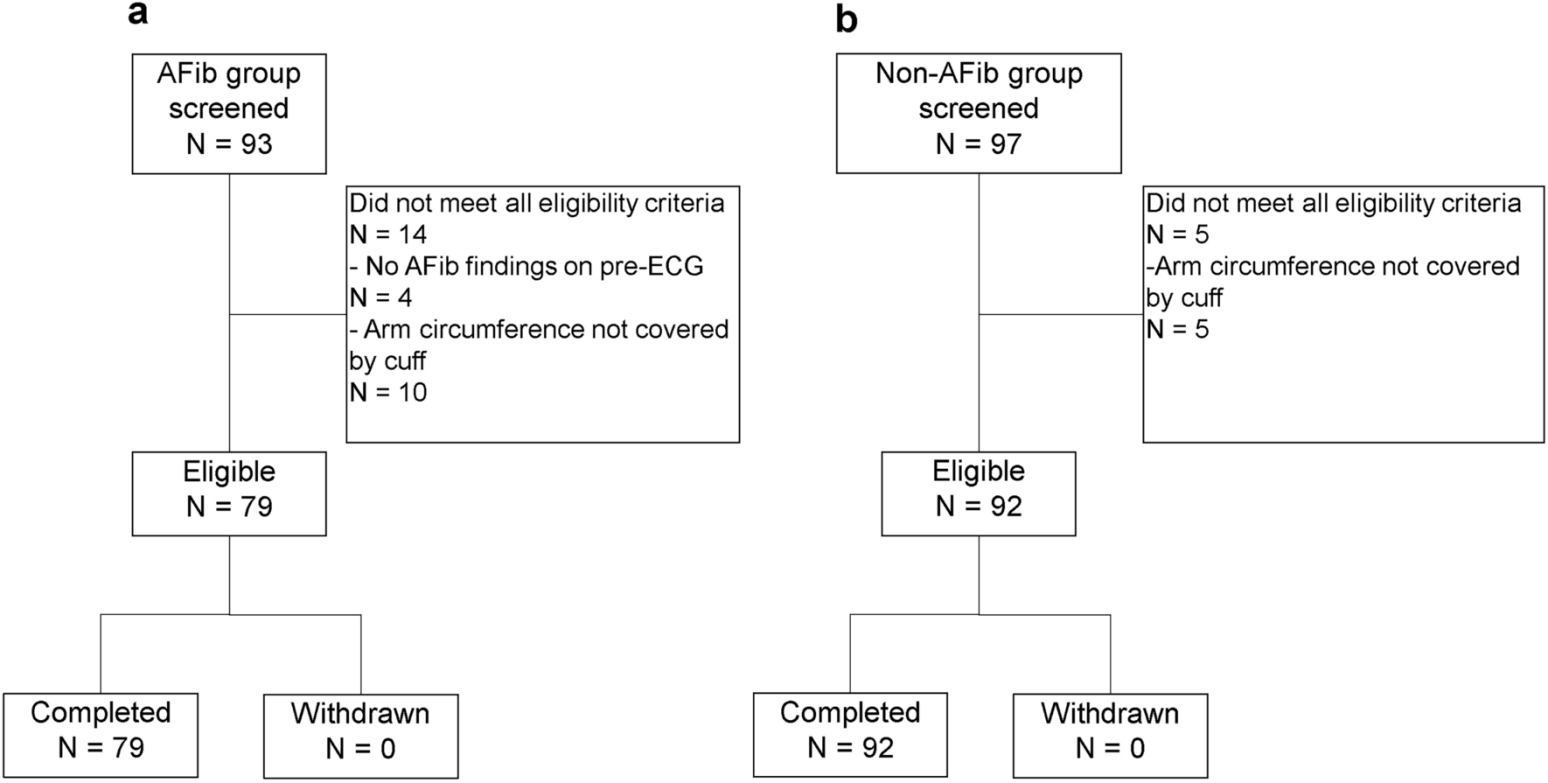
Flow charts of participants. In the atrial fibrillation (AFib) group, 93 participants were screened and 79 participants were measured completed (**a**). In the non-AFib group, 97 participants were screened and 92 participants were measured completed (**b**). *ECG* electrocardiogram.

Initially, 190 participants were screened, and 171 individuals who met the eligibility criteria were enrolled (Fig. 2a and Fig. 2b). The most common reason for exclusion was an upper arm circumference outside the study range. Other reasons included the absence of AFib symptoms on the ECG during the testing day in the AFib group. Table 1 shows the participants’ demographic characteristics. The median age of the AFib and non-AFib groups was 75 (50–92) years and 67 (24–87) years, respectively. Meanwhile, the median upper arm circumference of the AFib and non-AFib groups was 27.0 (22.0–35.0) cm and 27.0 (22.0–35.0) cm, respectively.

**Table 1:** Background of participants.

	AFib (n = 79)	non-AFib (n = 92)
Age (years)*	75 (50–92)	67 (24–87)
Gender (% of male)	69.6	69.6
Height (cm)*	165.0 (144.0–183.0)	167.5 (146.0–183.0)
Weight (kg)*	65.0 (43.7–95.1)	61.0 (41.0–93.0)
Upper arm circumference (cm)*	27.0 (22.0–35.0)	27.0 (22.0–35.0)

*AFib* atrial fibrillation.

*Data are expressed as median (range).

### Dataset for algorithm validation

A total of 171 PPI and ECG datasets were obtained, comprising 79 AFib and 92 non-AFib datasets. Each dataset consists of a PPI and ECG during FM31 and FL31 measurements. Each dataset was obtained from each participant. No duplicate measurements were performed for any single participant. Moreover, no BPM or ECG errors occurred in the final measurement (under the condition of up to two times repeated measurements in case of a BPM error). Therefore, missing PPI or ECG data were not included in the dataset. Number of PPI obtained during single BP measurement using FM31 and FL31 were 21.6 ± 3.4 pulses and 21.9 ± 3.5 pulses, respectively. All ECG results were interpreted by cardiologists and classified into “AFib” or “not AFib.” The dataset did not contain unreadable ECG data.

**Fig. 3:**
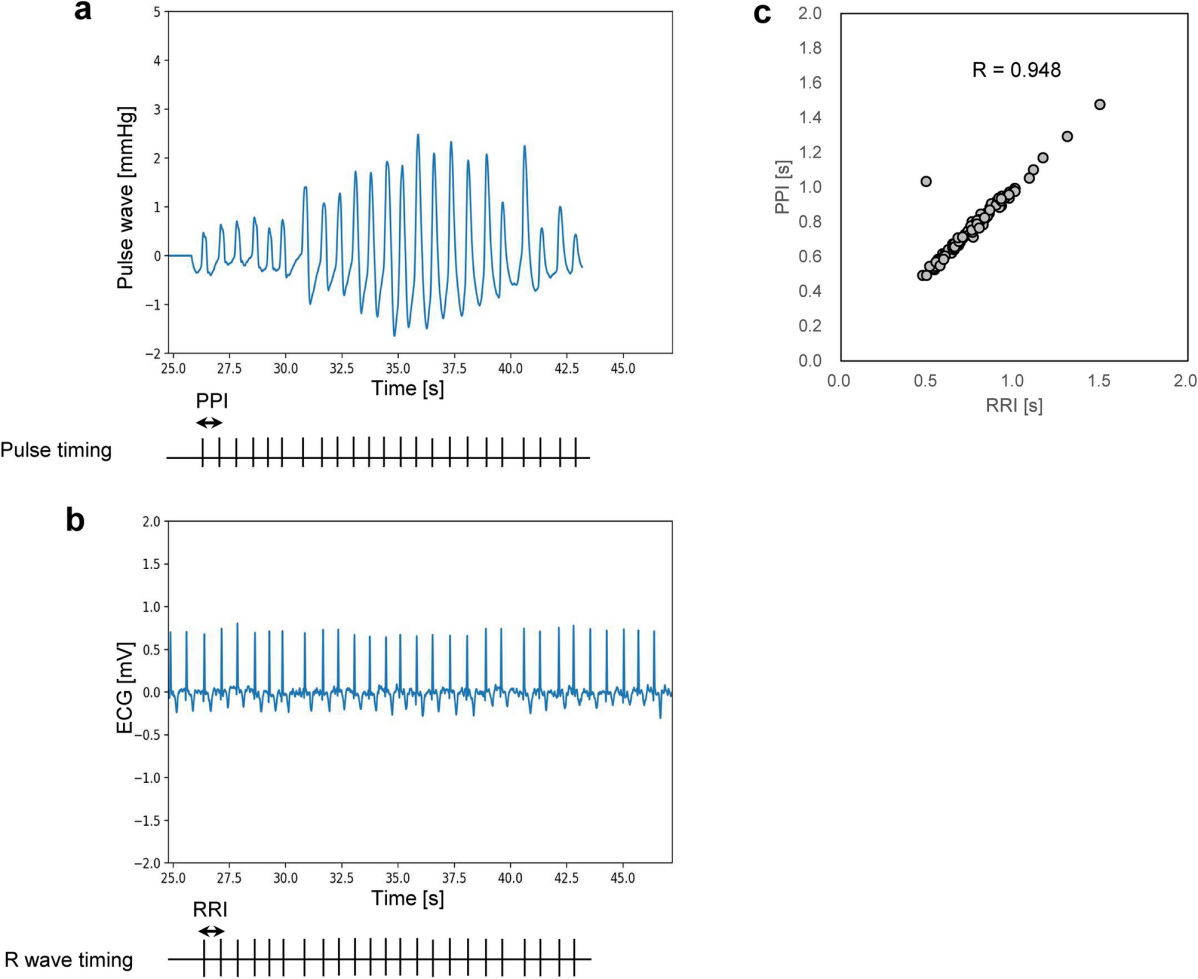
Association between the pulse-to-pulse intervals (PPIs) and the R-R intervals (RRI) by ECG in an AFib participant. The pressure pulse waves (PPWs) were measured by HEM-FM31 of blood pressure monitor in an AFib participant (**a**). The ECG waves were measured ECG monitor simultaneously (**b**). The raster plots indicate the timing of pulses peak (**a**) or R wave peak (**b**), these intervals indicate PPIs or RRIs. These figures show that the PPIs and RRIs patterns are synchronized approximately. Scatter plot between PPIs and RRIs is shown, indicates highly correlation (R = 0.948) (**c**). *ECG* electrocardiogram.

An example of PPW and ECG of AFib is shown in Fig. 3a and Fig. 3b. The pulse pattern of each PPW is synchronized with the R wave pattern of each corresponding ECG. Analysis of dataset from the last five participants with AFib showed that the PPI measured by the BPM highly correlated with the corresponding RRI (R = 0.948) (Fig. 3c). However, one PPI was not equal to the corresponding RRI because the pulse amplitude temporarily decreased, leading to skipping pulse detection by the pulse detector in the BPM. The results of our AFib algorithm were correct for all five participants despite including PPI with the outlier.

**Fig. 4:**
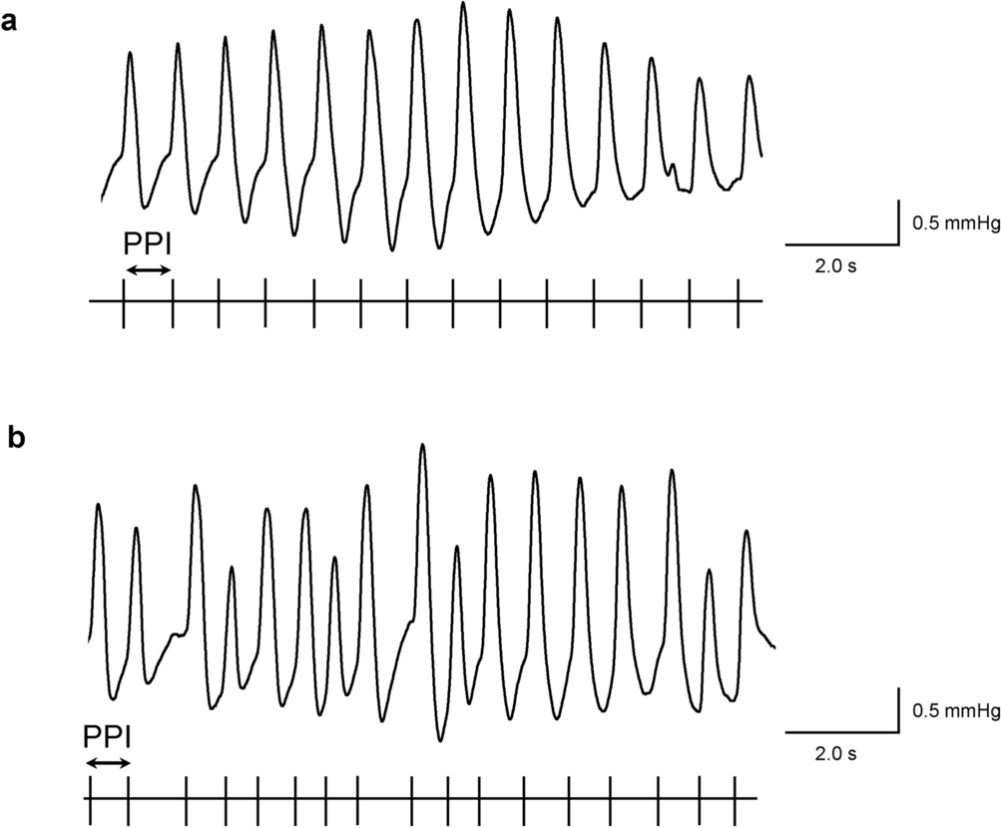
Representative pulse waves of normal sinus rhythm and atrial fibrillation (AFib). The pulse waves on the figure show a part of the entire pulse waves during blood pressure (BP) measurement. The raster plots indicate the timing of pulses. The pulse-to-pulse intervals (PPIs) are calculated by differentiating each pulse timings. This figure exhibits different PPI patterns in normal sinus rhythm (**a**) and AFib (**b**): constant PPIs in normal sinus rhythm and irregular PPIs in AFib.

The PPI in normal sinus rhythm was consistent and at a constant interval throughout the measured period (Fig. 4a). On the other hand, the PPI of AFib were irregular and random throughout the measured period (Fig. 4b). These examples show the differences in the PPI pattern between normal sinus rhythm and AFib.

### Performance of the AFib algorithm

The sensitivity of our AFib algorithm was 97.5% (95% CI 91.2–99.3) in the main-device (FM31) PPI dataset (Table 3). Two false-negative results were observed among the 79 AFib PPIs (Table 2). Both exhibited a temporary regular rhythm during BP measurements. Conversely, the specificity was 98.9% (95% CI 94.1–99.8) (Table 3). One false-positive result was observed among the 92 non-AFib PPIs (Table 2). A high-frequent PCs was observed in the ECGs with the false-positive PPI.

**Table 2:** Algorithm outputs for the AFib and non-AFib PPI data obtained by FM31 and FL31.

Algorithm output	AFib PPIs by FM31 (n = 79)	non-AFib PPIs by FM31 (n = 92)	AFib PPIs by FL31 (n = 79)	non-AFib PPIs by FL31 (n = 92)
AFib	77	1	74	0
Not AFib	2	91	5	92

FM31, OMRON HCR-7501T main unit and HEM-FM31 cuff; FL31, OMRON HEM7342T-Z main unit and HEM-FL31 cuff; *AFib* atrial fibrillation.

**Table 3:** Comparison of the performance between two different cuffs.

	FM31 (95% CI)	FL31 (95% CI)	*P*-Value*
Sensitivity (%)	97.5 (91.2–99.3)	93.7 (86.0–97.3)	0.26
Specificity (%)	98.9 (94.1–99.8)	100 (96.0–100)	0.32

*Statistical analyses were performed using the McNemar test.

*CI* confidence interval; FM31, OMRON HCR-7501T main unit and HEM-FM31 cuff; FL31, OMRON HEM7342T-Z main unit and HEM-FL31 cuff.

### Influence of cuff type on the performance of the AFib algorithm

Similar results were obtained in the sub-device (FL31) dataset. The sensitivity and specificity of our AFib algorithm were 93.7% (95% CI 86.0–97.3) and 100% (95% CI 96.0–100) in the FL31 PPI dataset, respectively (Table 3). No significant differences in the sensitivity (*P* = 0.26) and specificity (*P* = 0.32) were observed between the FM31 and FL31 groups (Table 3), suggesting that cuff size did not affect the accuracy of AFib detection. Five false-negative and zero false-positive results were observed respectively. Temporary regular heartbeats were also observed in the false-negative PPIs.

### Influence of age on the performance of the AFib algorithm

In the subgroup analysis by age, no statistically significant differences in sensitivity were observed between participants aged < 65 years and those aged ≥ 65 years (100% vs. 96.9%, *P* = 0.49) (Table 4). Moreover, no significant differences in specificity were observed between both groups (100% vs. 98.3%, *P* = 0.44), suggesting that age did not impact the performance of AFib detection (Table 4).

**Table 4:** Results of subgroup analysis regarding age and upper arm circumference.

	Age < 65 (15 in AFib, 34 in nonAFib)	Age ≥ 65 (64 in AFib, 58 in nonAFib)	*P*-Value*	Upper arm circumference < 27 (31 in AFib, 42 in nonAFib)	Upper arm circumference ≥ 27 (48 in AFib, 50 in nonAFib)	*P*-Value*
Sensitivity (%) (95% CI)	100 (79.6–100)	96.9 (89.3–99.1)	0.49	96.8 (83.8–99.4)	97.9 (89.1–99.6)	0.75
Specificity (%) (95% CI)	100 (90.0–100)	98.3 (90.9–99.7)	0.44	100 (91.6–100)	98.0 (89.5–99.6)	0.36

*Statistical analyses were performed using the chi-sqare test.

*AFib* atrial fibrillation; *CI* confidence interval.

### Influence of arm circumference on the performance of the AFib algorithm

In the subgroup analysis by upper arm circumference, no significant differences in sensitivity were observed between the small (< 27 cm) and large (≥ 27 cm) upper arm circumference groups (96.8% vs. 97.9%, *P* = 0.75) (Table 4). Moreover, no significant differences in specificity were observed between both groups (100% vs. 98.0%, *P* = 0.36) (Table 4), suggesting that upper arm circumference did not affect the performance of AFib detection within the cuff’s applicable range.

## Discussion

The sensitivity and specificity of our AFib algorithm using the PPI from a BPM were equivalent to those in the preliminary verification using heartbeat data of ECG dataset, even with a PPI dataset, suggesting the absence of overfitting. This PPI dataset had not been used previously during algorithm training and the algorithm had a high enough performance to detect AFib. Moreover, no statistically significant differences in sensitivity and specificity were observed during the subgroup analysis between different cuff types, age groups (< 65 and ≥ 65 years), and upper arm circumferences (< 27 and ≥ 27 cm). Therefore, the device type and individual’s age and upper arm circumference did not affect the performance of our AFib algorithm. These results indicate the feasibility of our algorithm for AFib screening using BPM. To the best of our knowledge, this study is the first to demonstrate the efficacy of a machine-learning-based algorithm for AFib detection using BPM PPIs.

We verified the absence of overfitting and achieved sensitivity and specificity results that were equivalent to those in the preliminary verification, even when studying clinical PPI dataset. Moreover, no statistically significant differences in sensitivity and specificity were observed during subgroup analysis between the two cuff types (FM31 and FL31), the two age groups (< 65 and ≥ 65 years), or the two upper arm circumferences (< 27 and ≥ 27 cm). These findings demonstrate that accuracy is unaffected by cuff type, age, or upper arm circumference when it is within the cuff adaptable range.

The PPI patterns recorded by the BPMs were synchronized and showed a high correlation to RRI (R = 0.948) (Fig. 3c). This result is consistent with previous studies by Ishizawa et al.^16^ and Kabutoya et al.^17^. Conversely, one outlier of PPI was observed since the pulse peak corresponding to the R peak of ECG temporarily decreased in PPW. However, our algorithm demonstrated high sensitivity even when such a pulse outlier was present, and our algorithm was confirmed to be robust against a certain degree of pulse outlier. This may be because the characteristics of C_STD_ were effective. C_STD_ has the characteristic of high noise tolerance. Miss-detection or over-detection of PPI causes a blend of longer or shorter intervals to happen, but such intervals are decomposed into an independent cluster. Therefore, C_STD_ cluster remains small although such outliers are blended into PPI. Hence, we identified that PPI and RRI can be treated as substantially equivalent. As previously mentioned, we trained our AFib algorithm using the RRI of ECGs, and using ECG data for training the PPI-based AFib algorithm is reasonable because the RRI has a pattern synchronized to the PPI of AFib. This analysis has two limitations. First, since ECG and PPW were measured using separate devices and had to be synchronized manually, which limited the number of synchronized datasets in this study. Hence, data analysis using the synchronized system is a future work. Second, the extent of pulse outliers without affecting performance remains unclear, so this analysis is a future work.

The deviation between PPW and PPI was found to be minimal compared to the fluctuation of PPI due to AFib. According to Wiesel et al.^10^, the fluctuation of the PPI due to AFib had an average irregularity index of 0.13. The irregularity index was defined as the standard deviation of the pulse intervals divided by the mean of the intervals for the total number of beats analyzed. Assuming an average heart rate of 80 bpm, the standard deviation of PPI in AFib can be calculated to 97.5 ms, where as the fluctuation of obtained PPI by our BPMs under constant artificial PPW was six fold lower than in AFib fluctuation. We therefore concluded that the accuracy of PPI does not inhibit the detection of AFib.

Since the 2000s, several attempts to achieve AFib detection by BPM have been reported^8^. Wiesel et al. first demonstrated the feasibility of AFib detection using BPM. They established a novel index for AFib detection based on PPI irregularity obtained using BPMs^10,12^, and this method showed sensitivity and specificity values of 96.8% and 88.8%, respectively^12^. Their AFib detection technology has been used in Microlife BPMs (Microlife Corporation, Clearwater, FL) with an AFib detection function. For example, Microlife BP3MQ1-2D showed sensitivity and specificity values of 99.2% and 92.9%, respectively^13^, whereas Microlife WatchBP Home A showed sensitivity and specificity values of 80.6% and 98.7%, respectively^27^. These Microlife BPMs are approved by the FDA and commercially available as prescription medical equipment. Ishizawa et al. also established a novel index for AFib detection based on an irregular heartbeat obtained using an OMRON BPM (HEM-907, OMRON HEALTHCARE), with a reported sensitivity and specificity of 95.5% and 96.5%, respectively^16^. Kabutoya et al. also established their original algorithm using an irregular heartbeat. They used the A&D BPM (UA-1020, A&D, Tokyo, Japan) and obtained irregular heartbeat, which showed 100% sensitivity and specificity^17^.

However, the conventional AFib detection technologies using PPI from BPM require three consecutive measurements for AFib detection while maintaining high accuracy. Our new AFib detection algorithm requires only a single measurement and has demonstrated the capability to make accurate determinations. Moreover, the sensitivity and specificity values of our AFib algorithm were 97.5% and 98.9%, respectively, which were comparable to those of the previously reported AFib detection algorithms (Table 5). This may have been achieved through the use of machine learning approach.

**Table 5:** Comparison of the performance between our study and studies of on AFib detection using BPM.

First author and year of publication	Blood pressure measuring device	Sensitivity (%) (95% CI)	Specificity (%) (95% CI)
Present study	Modified OMRON HCR-7501T	97.5 (91.2–99.3)	98.9 (94.1–99.8)
Wiesel 2009^10^	Modified Microlife BP3MQ1-2D	96.8 (91–99)	88.8 (85–92)
Wiesel 2013^11^	Microlife BP3MQ1-2D	99.2 (93.7–100)	92.9 (92.3–93.4)
Chan 2017^27^	Microlife WatchBP Home A	80.6 (69.5–88.9)	98.7 (98.3–98.9)
Ishizawa 2019^14^	Modified OMRON HEM-907	95.5 (84.0–99.6)	96.5 (93.4–98.3)
Kabutoya 2017^15^	Modified A&D UA-1020	100 (–)*	100 (–)*

*CI was not provided.

*AFib* atrial fibrillation, *CI* confidence interval.

In addition, performance of our AFib algorithm was comparable to those of wearable devices with ECG-based AFib detection algorithms. Recently, wearable devices with an ECG-based AFib detection feature have become commercially available. For example, the KardiaMobile has a reported sensitivity and specificity of 95% and 96%, respectively^28^. Following the KardiaMobile, several smart watches with AFib detection feature have received FDA clearance. These include the Apple Watch ECG app and Samsung Galaxy Watch (Samsung Electronics Co., Ltd., Suwon City, South Korea), which have a reported sensitivity of 93% and 95% and specificity of 96% and 93%, respectively^28^.

While these wearable devices have many advantages including the ability to measure the PPI anytime and anywhere, they are frequently designed for younger people and may be more difficult for older people to use. Our primary motivation is related to the convenience and utility of AFib screening, particularly for elderly and hypertensive patients, which are the two most significant risk groups. We believe that incorporating this screening feature into their daily BP measurement has the potential to provide early detection of AFib due to its ease of use.

Although this study demonstrated the high performance of our algorithm, it also shows false-negative and positive errors. When analyzing cases of classification error, we observed that the number of false negatives increased in AFib cases where the PPIs had a constant rhythm, resulting in decreased sensitivity. We believe that this limitation is because our algorithm considered only pulse wave intervals and did not take into account the absence of P-waves and the presence of fibrillation waves in the ECG, which are some of the main indications of AFib. Rare cases (e.g., AFib cases with complete atrioventricular block or atrial standstill) are thought to have constant heartbeats. However, these cases might have limited influence on AFib screening by BPMs because the main targets of screening^3^ are patients with early-stage AFib and those who do not recognize they have AFib. Atrial standstill is caused by long-standing AFib (late-stage AFib)^29^. In our study, false-positive errors were also observed in PPIs with frequent PCs. In patients with frequent PCs, especially multifocal PCs or PCs with bigeminal pulse patterns, the heartbeat rhythm tends to be irregular and random. PPIs infrequently show random heartbeats even those without AFib. This can create false positives because our AFib algorithm uses the heartbeat pattern. Similar cases have been reported in previous studies. For example, high-frequent PCs have been reported to lead to false-positive errors in BPM-based AFib detection^12,16^. However, we believe that this does not significantly impact the total performance of our AFib algorithm because this type of arrhythmia has a lower prevalence than normal sinus rhythm. This algorithm is also limited by the intermittent nature of AFib. If the patient is not in an active episode of AFib when the measurement is taken, no AFib will be detected. This limitation is common to all other algorithms in 12-lead ECG or mobile ECG devices. The detection of paroxysmal symptoms will be enhanced by daily or perhaps a bit more frequent testing^25^.

Number of PPIs obtained during single BP measurement using FM31 and FL31 were 21.6 ± 3.4 pulses and 21.9 ± 3.5 pulses, respectively. Assuming an average heart rate of 80 bpm, period of single BP measurement corresponds to 16.2 s and 16.4 s, respectively. The measurement period of wearable ECG device such as Apple watch and Kardia Mobile is approximately 30 s. Although performance of our AFib algorithm was comparable with these wearable devices, measurement period is shorter than these devices. In addition, the measurement period is not constant in BP measurement. It is not clear about relationship between the number of PPIs and performance in this study due to limited number of test data. In future, we will investigate minimum PPI value required for our AFib algorithm.

This study has two limitations. First, only Japanese participants were included, resulting in a limited number of individuals with an upper arm circumference of ≥ 35 cm in the dataset. Thus, we could not draw conclusions regarding individuals with thicker arms. Second, the actual BPM device embedded with AFib detection algorithm was not used in this study. Instead, a custom software on a personal computer was employed to judge AFib detection using the PPI dataset. Unexpected problems might occur when implementing it into a BPM device. Therefore, further studies are needed to verify the algorithm by implementing it on a BPM. Future studies involving large numbers of patients of other races and ethnicities will help to further validate the algorithm’s use on a global stage.

The main advantage of our AFib algorithm is that it can conduct AFib screening using a single BP measurement while maintaining high performance. Thus, AFib screening can be conducted during daily BP measurements without any special operation, enabling AFib screening to be performed without patients’ awareness. As mentioned in the introduction, older individuals with hypertension have a high risk of AFib^4,5^ and are the main users of BPMs. Senoo et al. also reported that daily AFib screening by home ECG leads to both earlier and more widespread detection of AFib than Holter ECG test and regular medical treatment^30^. In the future, we believe that BPMs equipped with our AFib algorithm can be a useful option for self screening for AFib.

## Conclusions

The goal of this study was to incorporate a reliable AFib screening into daily BP measurements. We successfully created an algorithm for AFib detection with machine learning technology using the PPI of a single BP measurement as input data. We also evaluated the performance of this AFib algorithm. The results showed that the sensitivity and specificity of the AFib algorithm were comparable to the conventional AFib detection algorithm with three BP measurements. This establishes the feasibility of this algorithm for AFib screening during daily BP measurements.

## Data Availability

Data cannot be shared publicly because this study involves human subjects. Minimal anonymized data are available from the corresponding author for researchers who meet the criteria for access to confidential data.
